# The Hydrophobia “Epidemic”

**Published:** 1899-09

**Authors:** 


A Monthly Review of Medicine and Surgery.

*EDITOR:*

WILLIAM WARREN POTTER, M. D.

All communications, whether of a literary or business nature, books for review and exchanges should be addressed to the editor: 284 FRANKLIN STREET, BUFFALO, N. Y.

VOL. XXXIX.—LV. SEPTEMBER, 1899. NO. 2.

### THE HYDROPHOBIA "EPIDEMIC."

FOR the past month the health department has had its hands full of what has been termed an "epidemic of hydrophobia." The events of the past thirty days giving rise to this condition may be summarised in a few words. In the latter part of July there was brought to the attention of the health commissioner, Dr. Wende, the case of a man who had been badly bitten by a dog which had run amuck through the district contiguous to Alden, in this county. That this dog was suffering with rabies when he attacked and bit the man there is no doubt. Its movements were closely followed from the time it left its owner's premises until it was killed in a nearby village, and it developed that it had bitten a large number of dogs, several other animals, and a number of human beings.

The very careful and painstaking investigation of this case brought to light many other cases wherein animals suspected of rabies had been killed. For a few days the matter rested and then cases of suspicious aspect came to light within the city limits. The health department took prompt action. To Dr. W. H. Heath, who by reason of experience was best qualified to report, was assigned the difficult task of investigation and diagnosis of cases within the city limits.

Dr. Wende issued a proclamation reciting the facts, and warning the public to keep dogs in off the streets. Complaints regarding sick dogs poured into the health department, and the many persons bitten by dogs during the month asked for investigation and opinion as to the conditions presented. Many undoubted cases of rabies in dogs were discovered. Where they had not done any damage they were taken to the pound and killed. Where animals under suspicion had bitten a human being, they were confined and kept under observation. In several cases undoubted rabies developed.

Up to the time of this writing there has been one death, that of a child at Sloan, just outside the city limits. This case was seen by Dr. Wende and Dr. Heath and was watched and closely studied for sometime before death.

The health department took hold of the matter promptly and acted decisively. The proclamation issued was clear cut and to the point, and the investigating has been thorough and searching.

It is problematical as yet what the outcome of all the dog bites reported will be in view of the uncertainty regarding the period of incubation. In the case of the child at Sloan between eighty and ninety days elapsed from the time the child was bitten to the appearance of the paroxysms.

Four patients were sent to the Pasteur Institute in New York City, at the expense of the county, and were returned home after treatment with assurances that they were practically safe from rabies.

One of the cases so treated was that of a man who was bitten by the same dog whose bite caused the death of the child at Sloan. His will be an interesting case to watch, since it is perfectly comparative. The dog was unquestionably mad. The child untreated by the Pasteur method died. The man treated lives. While there is no question regarding the efficacy of the treatment and the immense amount of good it has done, yet one cannot, from purely a scientific viewpoint, refrain from watching with an unusual degree of interest the outcome in this particular instance.

